# Modeling of the MET Sensitive Element Conversion Factor on the Intercathode Distance

**DOI:** 10.3390/s20185146

**Published:** 2020-09-09

**Authors:** Maksim Ryzhkov, Vadim Agafonov

**Affiliations:** Phystech School of Electronics, Photonics and Molecular Physics, Moscow Institute of Physics and Technology (National Research University), 141700 Dolgoprudny, Russia; maksim.ryzhkov@phystech.edu

**Keywords:** MET sensor, motion sensor, conversion factor, frequency response, electrochemical cell, diffusion, convection

## Abstract

MET sensors for measuring motion parameters are used in many scientific and technical fields. Meanwhile, the geometries of the transforming cell applied practically are far from optimal, and the influence of many geometric parameters on the sensitivity has not been studied. These parameters include the intercathode distance in a four-electrode conversion cell. In this paper, a mathematical model that allows calculating the behavior of the conversion coefficient depending on the frequency for a cell with flat electrodes at different intercathode distances is constructed. The stationary current is shown to decrease monotonically with the decreasing intercathode distance at the constancy of other system parameters. At the same time, the signal current decreases in the low-frequency region and increases in the high-frequency range. Taking into account the results obtained, practically speaking, it is advisable to reduce the intercathode distance to the technologically possible minimum, which makes the frequency response more uniform and reduces the current consumed by the sensitive element.

## 1. Introduction

The design of electrochemical motion sensors, also known as molecular-electronic transfer (MET) sensors, is based on a conversion element, which is an electrochemical cell made of several electrodes immersed in a channel filled with a highly conductive electrolyte solution [1,2]. Most often, a system of four electrodes is used. The electrodes are connected to the voltage source and located in the channel in the sequence anode–cathode–cathode–anode. The operation principle of this sensor is based on the dependence of the interelectrode current in the cell on the motion of the liquid in the specified channel. In turn, the movement of the liquid in the cell depends on the external mechanical influence. The main advantage of the MET sensors is high conversion rate of mechanical action into electrical signal. Initially, the MET-based sensors were used to measure low-frequency signals in seismometry [3,4,5,6]. Currently, the range of their application has expanded significantly and includes seismic exploration [7,8], hydroacoustics [9,10,11], monitoring of buildings and structures [12,13], medical applications [14], and inclinometry [15]. The reason for a noticeable expansion of applications is the development of new types of converting elements providing a significant expansion of the working frequency range in the high-frequency region.

The expansion of the frequency range posed new challenges for researchers. They are related to the fact that, for high frequencies, the absolute value of the conversion coefficient and its changes with frequency strongly depend on the micro-scale details of the converting element geometry, in particular, on the distance between the electrodes and their shape. At present, the effect on the conversion coefficient of a limited set of geometric parameters for a small number of possible configurations [16,17,18,19] has been studied experimentally and by simulation methods.

The influence of the intercathode distance on the MET sensors characteristics is a priori non-obvious and, therefore, the most interesting. Among currently known results, note the work in [20], where the conversion coefficient was proven to increase with the increasing intercathode distance. The effect is conditioned by the influence of the electric field on the charge transfer and is manifested only at the lowest frequencies, provided the cathode spacings are noticeably larger than the cathode–anode distance. In [21], the linearity of the signal conversion in relation to the intercathode distance was studied and nonlinear effects were shown to decrease with the decreasing distance. In addition, note that, from the known variety of configurations of the converting element, planar systems are of the most interest, as the electrodes in them are placed on the channels walls [19,22,23,24,25] due to the simplicity of manufacture and wide possibilities for selecting the converter geometry to achieve optimal output characteristics.

The present paper proposes a mathematical model for studying the frequency characteristics of the output signal of a planar molecular-electronic converter. The following model is used to study the frequency dependence of the sensitivity on the geometric parameters: the interelectrode distance and the size of the channel in for the fluid flow. In the entire frequency range, the conversion coefficient is found to be higher when the channel for fluid flow is narrower, and, therefore, when the main fluid flow is closer to the electrodes. The dependence of the sensitivity on the intercathode distance is more complex and depends on the signal frequency. At low frequencies, an increase in this distance leads to an increase in sensitivity, and vice versa at high frequencies. From a practical point of view, configurations with a shorter intercathode distance are preferable, since they provide a more uniform frequency response and the extension of the operating range in the high-frequency region, which is important for many applications.

## 2. Materials and Methods

In this paper, we consider an iodine–iodide electrochemical system, which is widely used in MET sensors [26,27]. The electrolyte in this system consists of one type of active ions and has a high concentration of the background component screening the field. An example of this scheme is a highly concentrated aqueous solution of potassium iodide KI or lithium iodide LiI or melted iodide salts mixed with molecular iodine $I_{2}$ in a small concentration. In excess of iodide, molecular iodine passes into triiodide, $I_{2}+I^{-}\to I_{3}^{-}$, which is the active component of the electrolyte solution.

In the presence of voltage on the electrodes, redox reactions occur on their surface: $I_{3}^{-}+2e\to 3I^{-}$ on the cathode and $3I^{-}-2e\to I_{3}^{-}$ on the anode. During the reactions on the cathode surface, the concentration of $I_{3}^{-}$ decreases, while on the anode surface it increases. As a result, a concentration gradient of the active component in the electrochemical cell is created. This results in convective diffusion of the active component of the electrolyte and consequent current emerge. When the applied voltage is sufficient for all the triiodide ions to react immediately upon reaching the cathode surface, saturation occurs, and the current in the system is determined by the delivery rate of the active component ions to the cathode surface. In this case, at the cathode, the concentration of the active component goes to zero. During a mechanical action on the system, the liquid comes in motion, which leads to a change in the rate of delivery of the active component to the cathode and the cathode current (which increases on one cathode and decreases on the other). The difference in the cathode currents (which equals zero in the absence of liquid motion due to the symmetry of the system) represents the response of the system to the external mechanical influence.

**Figure 1:**
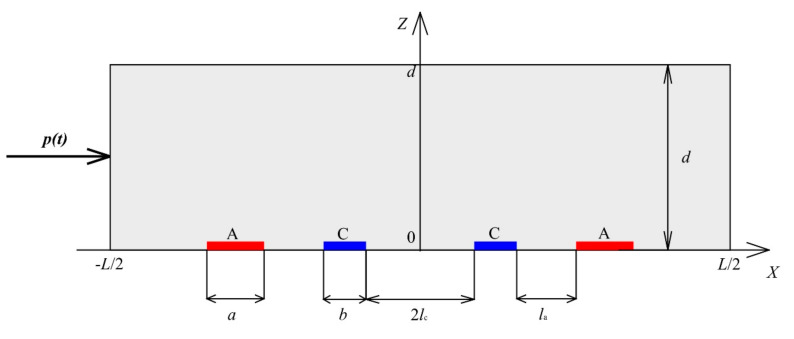
The circuit of the electrochemical cell with flat electrodes (A, anodes; C, cathodes).

The circuit of the electrochemical cell studied here with planar electrodes located on one of the channel walls is shown in Figure 1.

The channel has length L, thickness d, and width s, s≫L≫d. The crosswise size of the electrodes is equal to the channel width, the longitudinal size of the cathodes equals b, for the anodes it equals a, the distance between the cathodes and the anodes of one pair is $l_{a}$, and the distance between the cathodes is $2l_{c}$. The thickness of the electrodes is small compared to their crosswise and longitudinal dimensions.

The X-axis is directed along the channel, the Z-axis is perpendicular to the electrodes plane, and the Y-axis complements the right coordinate system. The origin of coordinates is in the center of the wall on which the electrodes are located.

Since the channel width is significantly larger than the remaining dimensions of the system, the system can be considered uniform throughout the channel width (along the Y-axis). That fact allows studying a flat channel, which is a section of the electrochemical cell by XZ plane.

Considering the electrolyte solution to be incompressible liquid, write the Navier–Stokes equations, the continuity equation describing the movement of the electrolyte solution, and the convective diffusion equation for the active component of the electrolyte, which describes the process of the convective ion transport of the active component between the electrodes in the electrochemical cell:

(1) $$\left\{\begin{array}{l} \frac{\partial v}{\partial t}=\nu \Delta v-\frac{1}{\rho }\nabla p\\ \mathrm{div}v=0\\ \frac{\partial c}{\partial t}=D\Delta c-\left(v\nabla \right)c \end{array}\right.,$$

where v is the velocity of the electrolyte solution in the channel, ν is the kinematic viscosity of the electrolyte solution, ρ is the density of the electrolyte solution, D is the diffusion coefficient of the active component of the electrolyte solution, and c is the concentration of the active component of the electrolyte solution.

Model the external mechanical signal effect on the liquid in the electrochemical cell by applying pressure to the left edge of the channel, which varies according to the harmonic law $p_{left}=p_{0}+p$, while, at the right end of the channel, the pressure is maintained unchanged $p_{right}=p_{0}$, where $p_{0}$ denotes the pressure at the ends of the channel in the absence of an external signal and $p=p_{\omega }e^{i\omega t}$ is the external signal (ω is the angular frequency of the external signal). As a result, at the ends of the channel, obtain the pressure difference that varies according to the harmonic law, which causes the flow of the electrolyte solution through the channel section.

As L≫d, then $v_{x}\gg v_{z}$. Considering the homogeneity of the system along the Y-axis, rewrite the Navier–Stokes equation and the continuity equation from Equation (1):

(2) $$\left\{\begin{array}{c} \frac{\partial v_{x}}{\partial t}=\nu \frac{\partial^{2}v_{x}}{\partial z^{2}}-\frac{1}{\rho }\frac{\partial p}{\partial x}=\nu \frac{\partial^{2}v_{x}}{\partial z^{2}}+\frac{p_{\omega }e^{i\omega t}}{\rho L}\\ \frac{\partial v_{x}}{\partial x}=0 \end{array}\right.,$$

where $\frac{\partial p}{\partial x}=-\frac{p_{left}-p_{right}}{L}=-\frac{p}{L}$.

The boundary conditions for the system in Equation (2) on the channel walls have the form:

(3) $$v_{x}\left(z=0\right)=v_{x}\left(z=d\right)=0.$$

The solution of Equation (2) is sought in the form: $v_{x}=v_{\omega x}e^{i\omega t}$. Taking into account the boundary conditions in Equation (3), obtain the expression for the x-component of the velocity of the electrolyte solution in the channel:

(4) $$v_{x}\left(z,t\right)=\frac{p}{i\omega \rho L\sinh\left(\alpha d\right)}\left(\sinh\left(\alpha d\right)-\sinh\left(\alpha z\right)-\sinh\left(\alpha d-\alpha z\right)\right),$$

where $\alpha =\sqrt{\frac{\omega }{2\nu }}\left(1+i\right)$.

Now, proceed to the solution of the convective diffusion equation from Equation (1). At low speeds of the electrolyte solution, the concentration of the active component can be shown as an expansion in powers of speed [22,28]:

(5) $$с={с}_{0}+{с}_{1},$$

where ${с}_{0}$ is the concentration of the active component in the stagnant electrolyte solution and ${с}_{1}$ is the linear in speed addition to the active component of the concentration. Substituting Equation (5) into the convective diffusion equation and discarding the terms of second and higher order of smallness, obtain:

(6) $$\left\{\begin{array}{l} \Delta c_{0}=0\\ \frac{\partial c_{1}}{\partial t}-D\Delta c_{1}=-\left(v\nabla \right)c_{0} \end{array}\right..$$

The solution of the second equation in Equation (6) is sought in the form: $c_{1}=c_{1\omega }e^{i\omega t}$, the index ω from $c_{1\omega }$ is omitted in further calculations. Applying the Fourier transform by x to Equation (6), and taking into account the homogeneity of the system along the Y-axis and $v_{x}\gg v_{z}$, obtain:

(7) $$\left\{\begin{array}{c} \begin{array}{c} \frac{\partial^{2}c_{0}\left(k,z\right)}{\partial z^{2}}-k^{2}c_{0}\left(k,z\right)=0 \end{array}\\ \frac{\partial^{2}c_{1}\left(k,z\right)}{\partial z^{2}}-\left(k^{2}+\frac{i\omega }{D}\right)c_{1}\left(k,z\right)=\frac{ikv_{x}}{D}c_{0}\left(k,z\right) \end{array}\right..$$

Obtain the boundary conditions for $c_{0,1}\left(k,z\right)$ from determining the current density on the electrodes surface:

(8) $$j_{0,1}\left(x\right)={\left.-Dq\frac{\partial c_{0,1}\left(x,0\right)}{\partial z}\right|}_{x\in S_{el}},$$

where q is the ion charge of the active component of the electrolyte solution.

Whence after applying the Fourier transform by x follows:

(9) $$\frac{\partial c_{0,1}\left(k,0\right)}{\partial z}=-\frac{j_{0,1}\left(k\right)}{Dq},$$

where $j_{0,1}\left(k\right)=\frac{1}{\sqrt{2\pi }}\int_{S_{el}}j_{0,1}\left(x\right)e^{-ikx}dx$ is the Fourier image of current density. $S_{el}$ denotes the electrodes surface.

Thus, the solution of the system in Equation (6) with the boundary conditions in Equation (9) has the form:

(10) $$c_{0}\left(k,z\right)=\frac{\cosh\left(kd-kz\right)}{\sinh\left(kd\right)}\frac{j_{0}\left(k\right)}{Dqk},$$

(11) $${с}_{1}\left(k,z\right)=\frac{\cosh\left(\beta d-\beta z\right)}{\sinh\left(\beta d\right)}\frac{j_{1}\left(k\right)}{Dq\beta }-\frac{\cosh\left(\beta d-\beta z\right)}{\beta }\int_{0}^{d}\frac{\cosh\left(\beta d-\beta \zeta \right)}{\sinh\left(\beta d\right)}\frac{ikv_{x}\left(\zeta \right)}{D}c_{0}\left(k,\zeta \right)d\zeta ++\frac{1}{\beta }\int_{0}^{d}\sinh\left(\beta z-\;-\beta \zeta \right)\frac{ikv_{x}\left(\zeta \right)}{D}c_{0}\left(k,\zeta \right)d\zeta ,$$

where $\beta =\sqrt{k^{2}+\frac{i\omega }{D}}$, Reβ>0.

After the inverse Fourier transform in Equations (10) and (11), obtain the expressions for the concentrations of the active component of the electrolyte on the plane z=0, on which the electrodes are located:

(12) $$c_{0}\left(x\right)=\frac{1}{2\pi Dq}\int_{-\infty }^{\infty }\frac{e^{ikx}dk}{k}\frac{\cosh\left(kd\right)}{\sinh\left(kd\right)}\int_{S_{el}}j_{0}\left(\xi \right)e^{-ik\xi }d\xi ,$$

(13) $$c_{1}\left(x\right)=\frac{1}{2\pi Dq}\int_{-\infty }^{\infty }\frac{e^{ikx}dk}{\beta }\frac{\cosh\left(\beta d\right)}{\sinh\left(\beta d\right)}\int_{S_{el}}j_{1}\left(\xi \right)e^{-ik\xi }d\xi -\;-\frac{1}{2\pi Dq}\frac{i}{D}\int_{-\infty }^{\infty }\frac{e^{ikx}dk}{\beta }\frac{\cosh\left(\beta d\right)}{\sinh\left(\beta d\right)}\int_{S_{el}}j_{0}\left(\xi \right)e^{-ik\xi }d\xi \int_{0}^{d}\frac{\cosh\left(\beta \zeta \right)}{\sinh\left(\beta d\right)}\frac{\cosh\left(k\zeta \right)}{\sinh\left(kd\right)}v_{x}\left(\zeta \right)d\zeta .$$

In the second term in Equation (11), a change is made: ζ→d−ζ. Moreover, it is considered that $v_{x}\left(\zeta \right)=v_{x}\left(d-\zeta \right)$.

Divide the electrodes into equal segments in width, while the current density within one segment has a constant value. Thus, the density of the stationary current $j_{0}$ and linear in the velocity current $j_{1}$ within the plane z=0 can be represented as:

(14) $$j_{0}\left(x\right)=\sum_{n=1}^{N}A_{0,n}\left(\theta \left(x-x_{n-1}^{a}\right)-\theta \left(x-x_{n}^{a}\right)+\theta \left(x+x_{n}^{a}\right)-\theta \left(x+x_{n-1}^{a}\right)\right)+\;+\sum_{n=1}^{N}B_{0,n}\left(\theta \left(x--x_{n-1}^{c}\right)-\theta \left(x-x_{n}^{c}\right)+\theta \left(x+x_{n}^{c}\right)-\theta \left(x+x_{n-1}^{c}\right)\right),$$

(15) $$j_{1}\left(x\right)=\sum_{n=1}^{N}A_{1,n}\left(\theta \left(x-x_{n-1}^{a}\right)-\theta \left(x-x_{n}^{a}\right)-\theta \left(x+x_{n}^{a}\right)+\theta \left(x+x_{n-1}^{a}\right)\right)+\;+\sum_{n=1}^{N}B_{1,n}\left(\theta \left(x--x_{n-1}^{c}\right)-\theta \left(x-x_{n}^{c}\right)-\theta \left(x+x_{n}^{c}\right)+\theta \left(x+x_{n-1}^{c}\right)\right),$$

where $x_{n}^{a}=l_{c}+b+l_{a}+\frac{n}{N}a$, $x_{n}^{c}=l_{c}+\frac{n}{N}b$, N is the number of segments, $A_{0,1n}$ are the current densities on the nth segment of the anode partition ($\left[x_{n-1}^{a},x_{n}^{a}\right]$), and $B_{0,1n}$ is the value of current densities on the nth segment of the cathode partition ($\left[x_{n-1}^{c},x_{n}^{c}\right]$). Equations (14) and (15) take into account that, due to the geometry of the system, the stationary current is an even function of x, and the current in Equation (15) linear in velocity is an odd function of x.

Placing Equations (14) and (15) into Equations (12) and (13), obtain for the points on the electrodes’ surfaces:

(16) $$\frac{4}{\pi Dq}\int_{0}^{\infty }dk\frac{\cos\left(kx\right)}{k^{2}}\frac{\cosh\left(kd\right)}{\sinh\left(kd\right)}\sum_{n=1}^{N}A_{0,n}\sin\left(\frac{ka}{2N}\right)\cos\left(k\frac{x_{n}^{a}+x_{n-1}^{a}}{2}\right)+\;+\frac{4}{\pi Dq}\int_{0}^{\infty }dk\frac{\cos\left(kx\right)}{k^{2}}\frac{\cosh\left(kd\right)}{\sinh\left(kd\right)}\sum_{n=1}^{N}B_{0,n}\sin\left(\frac{kb}{2N}\right)\cos\left(k\frac{x_{n}^{c}+x_{n-1}^{c}}{2}\right)=\left\{\begin{array}{c} c_{a},x\in anode\\ 0,x\in cathode \end{array}\right.,$$

(17) $$\frac{4}{\pi Dq}\int_{0}^{\infty }dk\frac{\sin\left(kx\right)}{\beta k}\frac{\cosh\left(\beta d\right)}{\sinh\left(\beta d\right)}\sum_{n=1}^{N}A_{1,n}\sin\left(\frac{ka}{2N}\right)\sin\left(k\frac{x_{n}^{a}+x_{n-1}^{a}}{2}\right)+\;+\frac{4}{\pi Dq}\int_{0}^{\infty }dk\frac{\sin\left(kx\right)}{\beta k}\frac{\cosh\left(\beta d\right)}{\sinh\left(\beta d\right)}\sum_{n=1}^{N}B_{1,n}\sin\left(\frac{kb}{2N}\right)\sin\left(k\frac{x_{n}^{c}+x_{n-1}^{c}}{2}\right)+\;+\frac{4}{\pi Dq}\frac{1}{D}\int_{0}^{\infty }dk\frac{\sin\left(kx\right)}{\beta k}\sum_{n=1}^{N}A_{0,n}\sin\left(\frac{ka}{2N}\right)\cos\left(k\frac{x_{n}^{a}+x_{n-1}^{a}}{2}\right)\int_{0}^{d}\frac{\cosh\left(\beta \zeta \right)}{\sinh\left(\beta d\right)}\frac{\cosh\left(k\zeta \right)}{\sinh\left(kd\right)}v_{x}\left(\zeta \right)d\zeta \;+\frac{4}{\pi Dq}\frac{1}{D}\int_{0}^{\infty }dk\frac{\sin\left(kx\right)}{\beta k}\sum_{n=1}^{N}B_{0,n}\sin\left(\frac{kb}{2N}\right)\cos\left(k\frac{x_{n}^{c}+x_{n-1}^{c}}{2}\right)\int_{0}^{d}\frac{\cosh\left(\beta \zeta \right)}{\sinh\left(\beta d\right)}\frac{\cosh\left(k\zeta \right)}{\sinh\left(kd\right)}v_{x}\left(\zeta \right)d\zeta =0,x\in S_{el}.$$

Here, $c_{a}$ denotes the concentration of the active component of the electrolyte solution on the surface of the anodes.

Now, turn to dimensionless quantities: $\tilde{b}=1$, $\tilde{a}=a/b$, $\tilde{d}=d/b$, $\tilde{L}=L/b$, ${\tilde{l}}_{c,a}=l_{c,a}/b$, $\tilde{x}=x/b$, ${\tilde{x}}_{n}^{a,c}=x_{n}^{a,c}/b$, $\tilde{z}=z/b$, $\tilde{\xi }=\xi /b$, $\tilde{\zeta }=\zeta /b$,$\;{\tilde{c}}_{a}=1$, ${\tilde{c}}_{0,1}\left(\tilde{x},\;\tilde{z}\right)=c_{0,1}\left(x,z\right)/c_{a}$, ${\tilde{j}}_{0,1}\left(\tilde{x}\right)=j_{0,1}\left(x\right)b/\left(Dqc_{a}\right)$, ${\tilde{A}}_{0,1,n}=A_{0,1,n}b/\left(Dqc_{a}\right)$, ${\tilde{B}}_{0,1,n}=B_{0,1,n}b/\left(Dqc_{a}\right)$, $\tilde{k}=kb$, $\tilde{\beta }=\beta b=\sqrt{{\tilde{k}}^{2}+i\tilde{\omega }}$, $\tilde{\omega }=\omega D/b^{2}$, $\tilde{f}=fD/b^{2}$, ${\overset{\text{˜}}{v}}_{\overset{\text{˜}}{x}}\left(\tilde{z}\right)=v_{x}\left(z\right)b/D$, where $f=\omega /\left(2\pi \right)$ is the frequency of external signal

After replacing the quantities with dimensionless ones in Equations (16) and (17), obtain:

(18) $$\frac{4}{\pi }\int_{0}^{\infty }d\tilde{k}\frac{\cos\left(\tilde{k}\tilde{x}\right)}{{\tilde{k}}^{2}}\frac{\cosh\left(\tilde{k}\tilde{d}\right)}{\sinh\left(\tilde{k}\tilde{d}\right)}\sum_{n=1}^{N}{\tilde{A}}_{0,n}\sin\left(\frac{\tilde{k}\tilde{a}}{2N}\right)\cos\left(\tilde{k}\frac{{\tilde{x}}_{n}^{a}+{\tilde{x}}_{n-1}^{a}}{2}\right)+\;+\frac{4}{\pi }\int_{0}^{\infty }d\tilde{k}\frac{\cos\left(\tilde{k}\tilde{x}\right)}{{\tilde{k}}^{2}}\frac{\cosh\left(\tilde{k}\tilde{d}\right)}{\sinh\left(\tilde{k}\tilde{d}\right)}\sum_{n=1}^{N}{\tilde{B}}_{0,n}\sin\left(\frac{\tilde{k}\tilde{b}}{2N}\right)\cos\left(\tilde{k}\frac{{\tilde{x}}_{n}^{c}+{\tilde{x}}_{n-1}^{c}}{2}\right)=\left\{\begin{array}{l} 1,\tilde{x}\in anode\\ 0,\tilde{x}\in cathode \end{array}\right.,$$

(19) $$\frac{4}{\pi }\int_{0}^{\infty }\frac{d\tilde{k}\sin\left(\tilde{k}\tilde{x}\right)}{\tilde{\beta }\tilde{k}}\frac{\cosh\left(\tilde{k}\tilde{d}\right)}{\sinh\left(\tilde{k}\tilde{d}\right)}\sum_{n=1}^{N}{\tilde{A}}_{1,n}\sin\left(\frac{\tilde{k}\tilde{a}}{2N}\right)\sin\left(\tilde{k}\frac{{\tilde{x}}_{n}^{a}+{\tilde{x}}_{n-1}^{a}}{2}\right)+\;+\frac{4}{\pi }\int_{0}^{\infty }\frac{d\tilde{k}\sin\left(\tilde{k}\tilde{x}\right)}{\tilde{\beta }\tilde{k}}\frac{\cosh\left(\tilde{k}\tilde{d}\right)}{\sinh\left(\tilde{k}\tilde{d}\right)}\sum_{n=1}^{N}{\tilde{B}}_{1,n}\sin\left(\frac{\tilde{k}\tilde{b}}{2N}\right)\sin\left(\tilde{k}\frac{{\tilde{x}}_{n}^{c}+{\tilde{x}}_{n-1}^{c}}{2}\right)+\;+\frac{4}{\pi }\int_{0}^{\infty }\frac{d\tilde{k}\sin\left(\tilde{k}\tilde{x}\right)}{\tilde{\beta }\tilde{k}}\sum_{n=1}^{N}{\tilde{A}}_{0,n}\sin\left(\frac{\tilde{k}\tilde{a}}{2N}\right)\cos\left(\tilde{k}\frac{{\tilde{x}}_{n}^{a}+{\tilde{x}}_{n-1}^{a}}{2}\right)\int_{0}^{\tilde{d}}\frac{\cosh\left(\tilde{\beta }\tilde{\zeta }\right)}{\sinh\left(\tilde{\beta }\tilde{d}\right)}\frac{\cosh\left(\tilde{k}\tilde{\zeta }\right)}{\sinh\left(\tilde{k}\tilde{d}\right)}{\overset{\text{˜}}{v}}_{\overset{\text{˜}}{x}}\left(\tilde{\zeta }\right)d\tilde{\zeta }+\;+\frac{4}{\pi }\int_{0}^{\infty }\frac{d\tilde{k}\sin\left(\tilde{k}\tilde{x}\right)}{\tilde{\beta }\tilde{k}}\sum_{n=1}^{N}{\tilde{B}}_{0,n}\sin\left(\frac{\tilde{k}\tilde{b}}{2N}\right)\cos\left(\tilde{k}\frac{{\tilde{x}}_{n}^{c}+{\tilde{x}}_{n-1}^{c}}{2}\right)\int_{0}^{\tilde{d}}\frac{\cosh\left(\tilde{\beta }\tilde{\zeta }\right)}{\sinh\left(\tilde{\beta }\tilde{d}\right)}\frac{\cosh\left(\tilde{k}\tilde{\zeta }\right)}{\sinh\left(\tilde{k}\tilde{d}\right)}{\overset{\text{˜}}{v}}_{\overset{\text{˜}}{x}}\left(\tilde{\zeta }\right)d\tilde{\zeta }=0,\tilde{x}\in S_{el}.$$

It can be noted that Equations (18) and (19) are the systems of linear equations relative to ${\tilde{A}}_{0,n}$, ${\tilde{B}}_{0,n}$ and ${\tilde{A}}_{1,n}$, ${\tilde{B}}_{1,n}$, respectively. In the matrix form, the system Equation (18) looks the following way:

(20) $$\left(\begin{array}{cc} R_{0}^{a} & S_{0}^{a}\\ R_{0}^{c} & S_{0}^{c} \end{array}\right)\left(\begin{array}{c} {\tilde{A}}_{0}\\ {\tilde{B}}_{0} \end{array}\right)=\left(\begin{array}{c} E\\ 0 \end{array}\right),$$

where

(21) $$R_{0,mn}^{a,c}=\frac{4}{\pi }\int_{0}^{\infty }\frac{d\tilde{k}}{{\tilde{k}}^{2}}\frac{\cosh\left(\tilde{k}\tilde{d}\right)}{\sinh\left(\tilde{k}\tilde{d}\right)}\cos\left(\tilde{k}\frac{{\tilde{x}}_{m}^{a,c}+{\tilde{x}}_{m-1}^{a,c}}{2}\right)\sin\left(\frac{\tilde{k}\tilde{a}}{2N}\right)\cos\left(\tilde{k}\frac{{\tilde{x}}_{n}^{a}+{\tilde{x}}_{n-1}^{a}}{2}\right),$$

(22) $$S_{0,mn}^{a,c}=\frac{4}{\pi }\int_{0}^{\infty }\frac{d\tilde{k}}{{\tilde{k}}^{2}}\frac{\cosh\left(\tilde{k}\tilde{d}\right)}{\sinh\left(\tilde{k}\tilde{d}\right)}\cos\left(\tilde{k}\frac{{\tilde{x}}_{m}^{a,c}+{\tilde{x}}_{m-1}^{a,c}}{2}\right)\sin\left(\frac{\tilde{k}\tilde{b}}{2N}\right)\cos\left(\tilde{k}\frac{{\tilde{x}}_{n}^{c}+{\tilde{x}}_{n-1}^{c}}{2}\right).$$

As $\tilde{x}$ in Equations (21) and (22) take the segmentation midpoints of the anodes and the cathodes, the coordinate of the middle of the mth segment can be written in the following form: $\tilde{x}=\frac{{\tilde{x}}_{m}^{a,c}+{\tilde{x}}_{m-1}^{a,c}}{2}$.

Note that the coefficients of the matrices $R_{0,mn}^{a,c}$ and $S_{0,mn}^{a,c}$ in Equations (21) and (22) are divergent integrals. That does not allow solving the system (20) in this form. Therefore, divide the integration gap $\left(0,\infty \right)$ in Equations (21) and (22) into two parts: $\left(0,h\right)$ and $\left(h,\infty \right)$, h>0. The integrals converge on the gap $\left(h,\infty \right)$ and diverge on the gap $\left(0,h\right)$.

Consider the gap $\left(0,h\right)$ in more detail. Expanding the integrands in Equations (21) and (22) in powers of k, obtain:

(23) $$\frac{1}{{\tilde{k}}^{2}}\frac{\cosh\left(\tilde{k}\tilde{d}\right)}{\sinh\left(\tilde{k}\tilde{d}\right)}\cos\left(\tilde{k}\frac{{\tilde{x}}_{m}^{a,c}+{\tilde{x}}_{m-1}^{a,c}}{2}\right)\sin\left(\frac{\tilde{k}\tilde{a}}{2N}\right)\cos\left(\tilde{k}\frac{{\tilde{x}}_{n}^{a}+{\tilde{x}}_{n-1}^{a}}{2}\right)=\frac{\tilde{a}}{2N\tilde{d}}\frac{1}{{\tilde{k}}^{2}}+\frac{\tilde{a}}{2N\tilde{d}}\left(\frac{{\tilde{d}}^{2}}{3}-\frac{{\tilde{a}}^{2}}{24N^{2}}-\;\frac{{\left({\tilde{x}}_{m}^{a,c}+{\tilde{x}}_{m-1}^{a,c}\right)}^{2}}{8}-\frac{{\left({\tilde{x}}_{n}^{a}+{\tilde{x}}_{n-1}^{a}\right)}^{2}}{8}\right)+ο\left({\tilde{k}}^{2}\right),$$

(24) $$\frac{1}{{\tilde{k}}^{2}}\frac{\cosh\left(\tilde{k}\tilde{d}\right)}{\sinh\left(\tilde{k}\tilde{d}\right)}\cos\left(\tilde{k}\frac{{\tilde{x}}_{m}^{a,c}+{\tilde{x}}_{m-1}^{a,c}}{2}\right)\sin\left(\frac{\tilde{k}\tilde{b}}{2N}\right)\cos\left(\tilde{k}\frac{{\tilde{x}}_{n}^{c}+{\tilde{x}}_{n-1}^{c}}{2}\right)=\frac{\tilde{b}}{2N\tilde{d}}\frac{1}{{\tilde{k}}^{2}}+\frac{\tilde{b}}{2N\tilde{d}}\left(\frac{{\tilde{d}}^{2}}{3}-\frac{{\tilde{b}}^{2}}{24N^{2}}-\;\frac{{\left({\tilde{x}}_{m}^{a,c}+{\tilde{x}}_{m-1}^{a,c}\right)}^{2}}{8}-\frac{{\left({\tilde{x}}_{n}^{c}+{\tilde{x}}_{n-1}^{c}\right)}^{2}}{8}\right)+ο\left({\tilde{k}}^{2}\right).$$

The integrals from the second and third terms of Equations (23) and (24) from $\left(0,h\right)$ converge, and, from the first terms diverge, however, after multiplying the first terms in Equations (23) and (24) by $A_{0n}$ and $B_{0n}$, respectively, and the subsequent summation, obtain:

$$\sum_{n=1}^{N}\left(\frac{1}{{\tilde{k}}^{2}}\frac{\tilde{a}}{2N\tilde{d}}{\tilde{A}}_{0n}+\frac{1}{{\tilde{k}}^{2}}\frac{\tilde{b}}{2N\tilde{d}}{\tilde{B}}_{0n}\right)=\frac{1}{2{\tilde{k}}^{2}\tilde{d}}\left(\sum_{n=1}^{N}\frac{\tilde{a}}{N}{\tilde{A}}_{0n}+\sum_{n=1}^{N}\frac{\tilde{b}}{N}{\tilde{B}}_{0n}\right)=\frac{1}{2{\tilde{k}}^{2}\tilde{d}}\left({\tilde{I}}_{0a}+{\tilde{I}}_{0c}\right)\equiv 0,$$

where ${\tilde{I}}_{0a}$ and $\;{\tilde{I}}_{0c}$ are the total current in the stagnant electrolyte solution at the anode and the cathode, respectively. The sum of these currents after establishing equilibrium in such solution is identically equal to zero because of the charge conservation law. Therefore, the diverging part in the integrals in Equations (21) and (22) leaves when summed over all segments. Consequently, in Equations (23) and (24), the first terms can be discarded. Thus, after the described transformations of the coefficients of the matrix of the system in Equation (20), obtain:

(25) $$\left(\begin{array}{cc} {\tilde{R}}_{0}^{a} & {\tilde{S}}_{0}^{a}\\ {\tilde{R}}_{0}^{c} & {\tilde{S}}_{0}^{c} \end{array}\right)\left(\begin{array}{c} {\tilde{A}}_{0}\\ {\tilde{B}}_{0} \end{array}\right)=\left(\begin{array}{c} E\\ 0 \end{array}\right),$$

where ${\tilde{A}}_{0}$ is the vector with components ${\tilde{A}}_{0n}$, ${\tilde{B}}_{0}$ is the vector with components ${\tilde{B}}_{0n}$, and $\left(\begin{array}{c} E\\ 0 \end{array}\right)$ is the vector in which the first N components are units, while the remaining components are zero,

$${\tilde{R}}_{0,mn}^{a,c}=\frac{2}{\pi }\int_{0}^{h}d\tilde{k}\frac{\tilde{a}}{N\tilde{d}}\left(\frac{{\tilde{d}}^{2}}{3}-\frac{{\tilde{a}}^{2}}{24N^{2}}-\frac{{\left({\tilde{x}}_{m}^{a,c}+{\tilde{x}}_{m-1}^{a,c}\right)}^{2}}{8}-\frac{{\left({\tilde{x}}_{n}^{a}+{\tilde{x}}_{n-1}^{a}\right)}^{2}}{8}\right)+\;\frac{4}{\pi }\int_{h}^{\infty }\frac{d\tilde{k}}{{\tilde{k}}^{2}}\frac{\cosh\left(\tilde{k}\tilde{d}\right)}{\sinh\left(\tilde{k}\tilde{d}\right)}\cos\left(\tilde{k}\frac{{\tilde{x}}_{m}^{a,c}+{\tilde{x}}_{m-1}^{a,c}}{2}\right)\sin\left(\frac{\tilde{k}\tilde{a}}{2N}\right)\cos\left(\tilde{k}\frac{{\tilde{x}}_{n}^{a}+{\tilde{x}}_{n-1}^{a}}{2}\right),\;{\tilde{S}}_{0,mn}^{a,c}=\frac{2}{\pi }\int_{0}^{h}d\tilde{k}\frac{\tilde{b}}{N\tilde{d}}\left(\frac{{\tilde{d}}^{2}}{3}-\frac{{\tilde{a}}^{2}}{24N^{2}}-\frac{{\left({\tilde{x}}_{m}^{a,c}+{\tilde{x}}_{m-1}^{a,c}\right)}^{2}}{8}-\frac{{\left({\tilde{x}}_{n}^{c}+{\tilde{x}}_{n-1}^{c}\right)}^{2}}{8}\right)+\;\frac{4}{\pi }\int_{h}^{\infty }\frac{d\tilde{k}}{{\tilde{k}}^{2}}\frac{\cosh\left(\tilde{k}\tilde{d}\right)}{\sinh\left(\tilde{k}\tilde{d}\right)}\cos\left(\tilde{k}\frac{{\tilde{x}}_{m}^{a,c}+{\tilde{x}}_{m-1}^{a,c}}{2}\right)\sin\left(\frac{\tilde{k}\tilde{b}}{2N}\right)\cos\left(\tilde{k}\frac{{\tilde{x}}_{n}^{c}+{\tilde{x}}_{n-1}^{c}}{2}\right).$$

Taking h small enough, a solution to the system in Equation (20) can be obtained with the required accuracy.

The matrix form Equation (19) is similar to Equation (20):

(26) $$\left(\begin{array}{cc} R_{1}^{a} & S_{1}^{a}\\ R_{1}^{c} & S_{1}^{c} \end{array}\right)\left(\begin{array}{c} {\tilde{A}}_{1}\\ {\tilde{B}}_{1} \end{array}\right)=\left(\begin{array}{cc} r^{a} & s^{a}\\ r^{c} & s^{c} \end{array}\right)\left(\begin{array}{c} {\tilde{A}}_{0}\\ {\tilde{B}}_{0} \end{array}\right),$$

where ${\tilde{A}}_{1}$ is the vector with components ${\tilde{A}}_{1,n}$ and ${\tilde{B}}_{1}$ is the vector with components ${\tilde{B}}_{1,n}$,

$$R_{1,mn}^{a,c}=\frac{4}{\pi }\int_{0}^{\infty }\frac{d\tilde{k}}{\tilde{\beta }\tilde{k}}\frac{\cosh\left(\tilde{\beta }\tilde{d}\right)}{\sinh\left(\tilde{\beta }\tilde{d}\right)}\sin\left(\tilde{k}\frac{{\tilde{x}}_{m}^{a,c}+{\tilde{x}}_{m-1}^{a,c}}{2}\right)\sin\left(\frac{\tilde{k}\tilde{a}}{2N}\right)\sin\left(\tilde{k}\frac{{\tilde{x}}_{n}^{a}+{\tilde{x}}_{n-1}^{a}}{2}\right),\;S_{1,mn}^{a,c}=\frac{4}{\pi }\int_{0}^{\infty }\frac{d\tilde{k}}{\tilde{\beta }\tilde{k}}\frac{\cosh\left(\tilde{\beta }\tilde{d}\right)}{\sinh\left(\tilde{\beta }\tilde{d}\right)}\sin\left(\tilde{k}\frac{{\tilde{x}}_{m}^{a,c}+{\tilde{x}}_{m-1}^{a,c}}{2}\right)\sin\left(\frac{\tilde{k}\tilde{b}}{2N}\right)\sin\left(\tilde{k}\frac{{\tilde{x}}_{n}^{c}+{\tilde{x}}_{n-1}^{c}}{2}\right),\;r_{mn}^{a,c}=-\frac{4}{\pi }\int_{0}^{\infty }\frac{d\tilde{k}}{\tilde{\beta }\tilde{k}}\sin\left(\tilde{k}\frac{{\tilde{x}}_{m}^{a,c}+{\tilde{x}}_{m-1}^{a,c}}{2}\right)\sin\left(\frac{\tilde{k}\tilde{a}}{2N}\right)\cos\left(\tilde{k}\frac{{\tilde{x}}_{n}^{a}+{\tilde{x}}_{n-1}^{a}}{2}\right)\int_{0}^{\tilde{d}}\frac{\cosh\left(\tilde{\beta }\tilde{\zeta }\right)}{\sinh\left(\tilde{\beta }\tilde{d}\right)}\frac{\cosh\left(\tilde{k}\tilde{\zeta }\right)}{\sinh\left(\tilde{k}\tilde{d}\right)}{\overset{\text{˜}}{v}}_{\overset{\text{˜}}{x}}\left(\tilde{\zeta }\right)d\tilde{\zeta },\;s_{mn}^{a,c}=-\frac{4}{\pi }\int_{0}^{\infty }\frac{d\tilde{k}}{\tilde{\beta }\tilde{k}}\sin\left(\tilde{k}\frac{{\tilde{x}}_{m}^{a,c}+{\tilde{x}}_{m-1}^{a,c}}{2}\right)\sin\left(\frac{\tilde{k}\tilde{b}}{2N}\right)\cos\left(\tilde{k}\frac{{\tilde{x}}_{n}^{c}+{\tilde{x}}_{n-1}^{c}}{2}\right)\int_{0}^{\tilde{d}}\frac{\cosh\left(\tilde{\beta }\tilde{\zeta }\right)}{\sinh\left(\tilde{\beta }\tilde{d}\right)}\frac{\cosh\left(\tilde{k}\tilde{\zeta }\right)}{\sinh\left(\tilde{k}\tilde{d}\right)}{\overset{\text{˜}}{v}}_{\overset{\text{˜}}{x}}\left(\tilde{\zeta }\right)d\tilde{\zeta }.$$

The solution of the systems Equations (25) and (26) is described by the following formulas:

(27) $$\left(\begin{array}{c} {\tilde{A}}_{0}\\ {\tilde{B}}_{0} \end{array}\right)={\left(\begin{array}{cc} {\tilde{R}}_{0}^{a} & {\tilde{S}}_{0}^{a}\\ {\tilde{R}}_{0}^{c} & {\tilde{S}}_{0}^{c} \end{array}\right)}^{-1}\left(\begin{array}{c} E\\ 0 \end{array}\right),$$

(28) $$\left(\begin{array}{c} {\tilde{A}}_{1}\\ {\tilde{B}}_{1} \end{array}\right)={\left(\begin{array}{cc} R_{1}^{a} & S_{1}^{a}\\ R_{1}^{c} & S_{1}^{c} \end{array}\right)}^{-1}\left(\begin{array}{cc} r^{a} & s^{a}\\ r^{c} & s^{c} \end{array}\right)\left(\begin{array}{c} {\tilde{A}}_{0}\\ {\tilde{B}}_{0} \end{array}\right).$$

## 3. Results

Since the output signal in this electrochemical cell is the difference of the cathode currents, only the current at the cathodes is considered further.

The dimensionless difference cathode current ${\tilde{J}}_{1}$, which is linear in speed, is calculated by the formula:

(29) $${\tilde{J}}_{1}=\int_{S_{cath1}}{\tilde{j}}_{1}\left(\tilde{x}\right)d\tilde{x}-\int_{S_{cath2}}{\tilde{j}}_{1}\left(\tilde{x}\right)d\tilde{x}=\frac{2\tilde{b}}{N}\sum_{n=1}^{N}{\tilde{B}}_{1,n},$$

where $S_{cath1}$ and $S_{cath2}$ are the surfaces of the left and right cathodes from the circuit in Figure 1, respectively.

Equation (29) describes the response of an electrochemical cell to an external signal.

For subsequent considerations, take the following parameters which are typical for practical electrochemical cells: $\tilde{b}=1$, $\tilde{a}=1$, ${\tilde{l}}_{a}=1$, $\tilde{L}=100$. For the $\tilde{d}$ parameter, take $\tilde{d}=2$, which can also be considered as close to optimal [24,29] because at lower $\tilde{d}$ the flow of the liquid will be difficult due to the large hydrodynamic resistance, while at large $\tilde{d}$ a significant part of the flow will be away from the electrodes, which also leads to a decrease in the signal conversion coefficient. In this case, the electrodes are divided into N=25 equal segments. Divide the frequencies into three regions: low (${\tilde{\lambda }}_{D}>\tilde{d}/2$), medium (${\tilde{\lambda }}_{D}∼\tilde{d}/2$), and high (${\tilde{\lambda }}_{D}\ll \tilde{d}/2$). ${\tilde{\lambda }}_{D}=\sqrt{1/\tilde{\omega }}=\sqrt{1/\left(2\pi \tilde{f}\right)}$ is the dimensionless diffusion length of the active component of the electrolyte solution (diffusion particle length is defined as: $\lambda_{D}=\sqrt{D/\omega }$).

### 3.1. Stationary Current Density at the Cathodes

**Figure 2:**
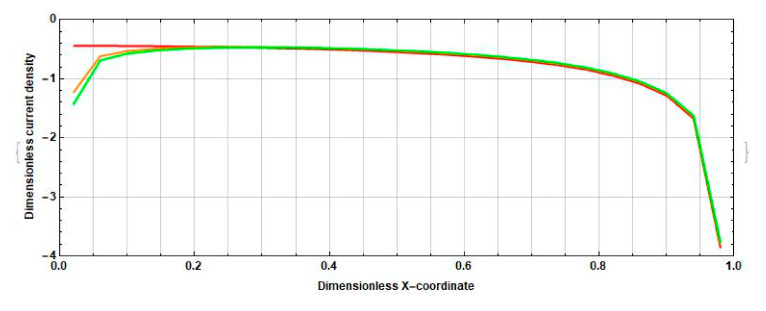
Distribution of stationary current density over the cathode surface at different distances between the cathodes (red line, $2{\tilde{l}}_{c}=0$; orange line, $2{\tilde{l}}_{c}=0.5$; green line, $2{\tilde{l}}_{c}=1$).

Compare the density distribution of the stationary cathode current on the cathode surface at different intercathode distances. For definiteness, consider the cathode, to the right of which there is an anode and to the left of which there is another cathode. In Figure 1, the right cathode corresponds to this. Due to the symmetry of the system and the consequent parity of the stationary current density along the X-axis for the other cathode, the distribution is the same. The corresponding distributions at $2{\tilde{l}}_{c}=0$, $2{\tilde{l}}_{c}=0.5$, $2{\tilde{l}}_{c}=1$ are shown in Figure 2.

The negative value of the density of the stationary current at the cathode is related to the form of the definition Equation (8) and expresses the physical fact that the stationary current flows from the anode to the cathode.

Figure 2 shows that the absolute value of the density of the stationary current decreases slightly in the middle part and in the part of the cathode closest to the adjacent anode. However, in the opposite part of the electrode, a significant increase (up to three times) in the absolute value of the density of the stationary current is observed, and the growth value decreases from the edge to the center of the cathode.

As a result, the total stationary cathode current increases with increasing distance between the cathodes.

### 3.2. Density of the Current Linear in Velocity at the Cathodes

**Figure 3:**
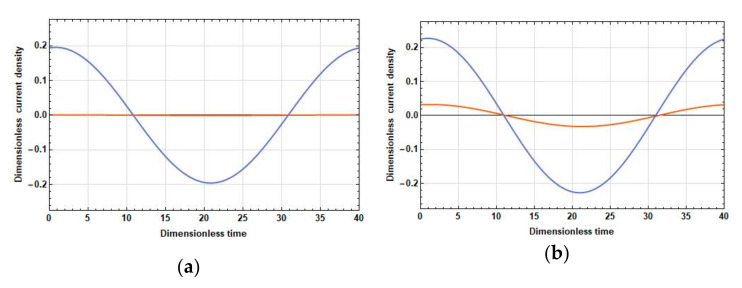
A change in time of a linear density current at the edges of the cathodes during the period of an external signal with the frequency $\tilde{f}=0.025$ at: (**a**) $2{\tilde{l}}_{c}=0$; and (**b**) $2{\tilde{l}}_{c}=1$. ${\tilde{B}}_{1,1}$ (orange lines) is the cathode edge from the side of the other cathode and ${\tilde{B}}_{1,25}$ (blue lines) is the cathode edge from the side of the adjacent anode.

**Figure 4:**
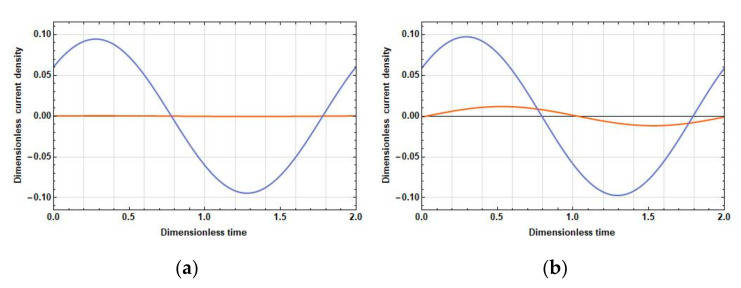
A change in time of a linear density current at the edges of the cathodes during the period of an external signal with the frequency $\tilde{f}=0.5$ at: (**a**) $2{\tilde{l}}_{c}=0$; and (**b**) $2{\tilde{l}}_{c}=1$. ${\tilde{B}}_{1,1}$ (orange lines) is the cathode edge from the side of the other cathode and ${\tilde{B}}_{1,25}$ (blue lines) is the cathode edge from the side of the adjacent anode.

**Figure 5:**
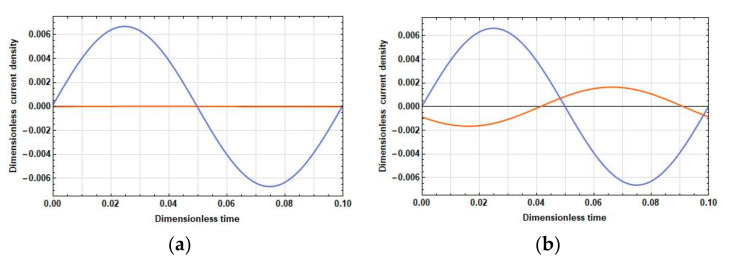
A change in time of a linear density current at the edges of the cathodes during the period of an external signal with the frequency $\tilde{f}=10$ at: (**a**) $2{\tilde{l}}_{c}=0$; and (**b**) $2{\tilde{l}}_{c}=1$. ${\tilde{B}}_{1,1}$ (orange lines) is the cathode edge from the side of the other cathode and ${\tilde{B}}_{1,25}$ (blue lines) is the cathode edge from the side of the adjacent anode.

The density of the current linear in velocity, unlike the stationary one, can significantly differ in phase in different parts of the electrodes. Therefore, compare the dependences of the density of the current linear in velocity on time at the opposite ends of the cathodes at $2{\tilde{l}}_{c}=0$, $2{\tilde{l}}_{c}=1$ and $\tilde{f}=0.025$ (${\tilde{\lambda }}_{D}=2.52$, low frequencies), $\tilde{f}=0.5$ (${\tilde{\lambda }}_{D}=0.56$, medium frequencies), and $\tilde{f}=10$ (${\tilde{\lambda }}_{D}=0.13$, high frequencies). The graphs of these dependences are given in Figure 3, Figure 4 and Figure 5.

Figure 3, Figure 4 and Figure 5 show that, in all frequency ranges, at zero distance between the cathodes, the amplitude ${\tilde{B}}_{1,1}$ is negligible compared to the amplitude ${\tilde{B}}_{1,25}$. As the distance between the cathodes increases, ${\tilde{B}}_{1,1}$ increases significantly; ${\tilde{B}}_{1,25}$ also increases, but not so significantly. Besides:

- In the field of low frequencies, ${\tilde{B}}_{1,1}$ and ${\tilde{B}}_{1,25}$ are practically in phase.
- In the medium range, the phase difference between ${\tilde{B}}_{1,1}$ and ${\tilde{B}}_{1,25}$ is already quite significant and is about π/5.
- At high frequencies, the oscillations ${\tilde{B}}_{1,1}$ are ${\tilde{B}}_{1,25}$ close to the opposite phase. Thus, from the expression for the response, the following conclusions have been made:
- With an increase in the distance between the cathodes, the difference contribution of the part adjacent to the cathode surface into the total current is positive due to an increase in the amplitude of the current density in this part and the proximity of the phases of the current densities in the parts adjacent to the cathode and the anode.
- At high frequencies, an increase in the amplitude of the current density in the part adjacent to the cathode leads to a decrease in the total current due to the phase difference between the parts adjacent to the anode and the cathode parts, which is close to π.

### 3.3. Electrochemical Cell Sensitivity

Define the sensitivity of the electrochemical cell $\tilde{W}$ as the ratio of the difference cathode current to the two-dimensional flow rate of the electrolyte solution through the channel cross section $\tilde{Q}$:

(30) $$\tilde{W}=\frac{{\tilde{J}}_{1}}{\tilde{Q}},$$

where

(31) $$\tilde{Q}=\int_{0}^{\tilde{d}}{\overset{\text{˜}}{v}}_{\overset{\text{˜}}{x}}\left(\tilde{z}\right)d\tilde{z}.$$

As can be seen from Equations (4) and (31), the two-dimensional flow rate is linear with respect to the external signal p, since ${\tilde{J}}_{1}$ is also linear in the external signal according to Equations (4) and (27)–(29). Thus, Equation (30) depends only on the frequency of the external signal and the parameters of the electrochemical cell and does not depend on the amplitude of the external signal.

#### 3.3.1. Dependence of the Sensitivity on the Intercathode Distance

**Figure 6:**
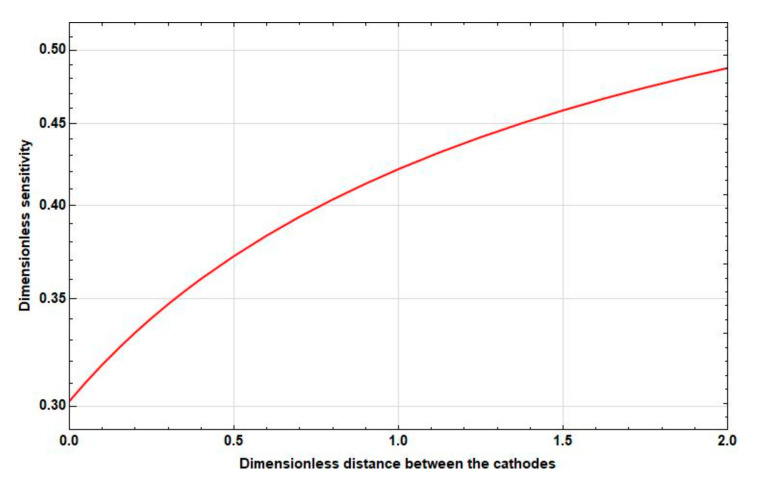
Dependence of the sensitivity on the intercathode distance at $\tilde{f}=0.025$.

**Figure 7:**
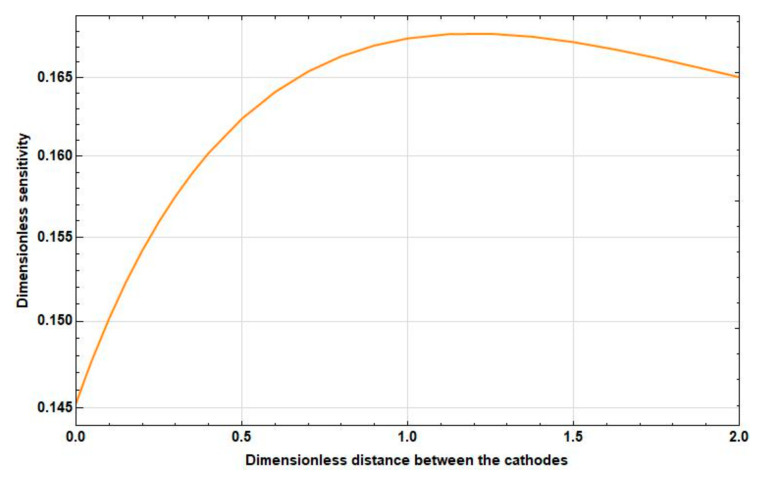
Dependence of the sensitivity on the intercathode distance at $\tilde{f}=0.5$.

**Figure 8:**
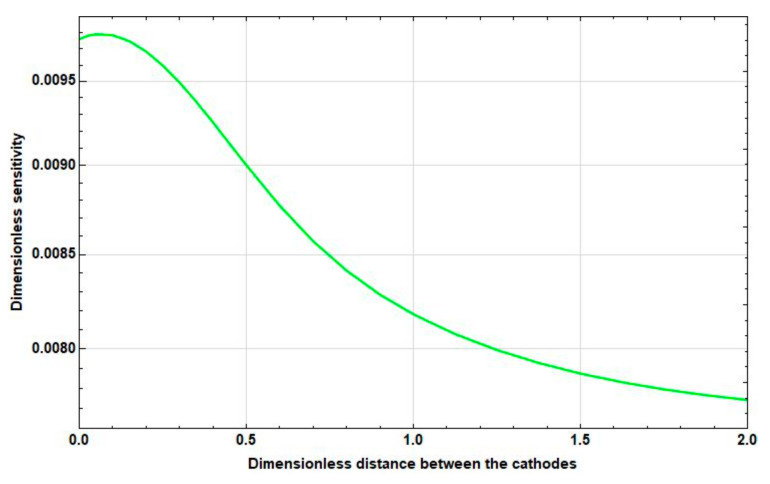
Dependence of the sensitivity on the intercathode distance at $\tilde{f}=10$.

Study the dependence of the sensitivity on the frequency and distance between the cathodes. The graphs of the corresponding dependences are presented in Figure 6, Figure 7 and Figure 8.

As Figure 6, Figure 7 and Figure 8 show, as the intercathode distance between the cathodes increases to certain values, an increase in sensitivity is observed in all frequency ranges. Wherein:

- At high frequencies, this increase is negligible and is replaced by a decrease already at small distances between the cathodes ($2{\tilde{l}}_{c}∼0.1$).
- At medium frequencies, the increase goes to the distance between the cathodes $2{\tilde{l}}_{c}\approx 1$. Then, the sensitivity starts to decrease. In this case, the maximum sensitivity is greater than the sensitivity at zero distance between the cathodes at 10–15%.
- At low frequencies, there is a significant, more than 50%, increase in sensitivity in the interval from $2{\tilde{l}}_{c}=0$ to $2{\tilde{l}}_{c}=2$, which continues even at $2{\tilde{l}}_{c}>2$.

#### 3.3.2. Dependence of the Sensitivity on the Frequency

Now, study the frequency dependence of the sensitivity of the electrochemical cell at various intercathode distances.

**Figure 9:**
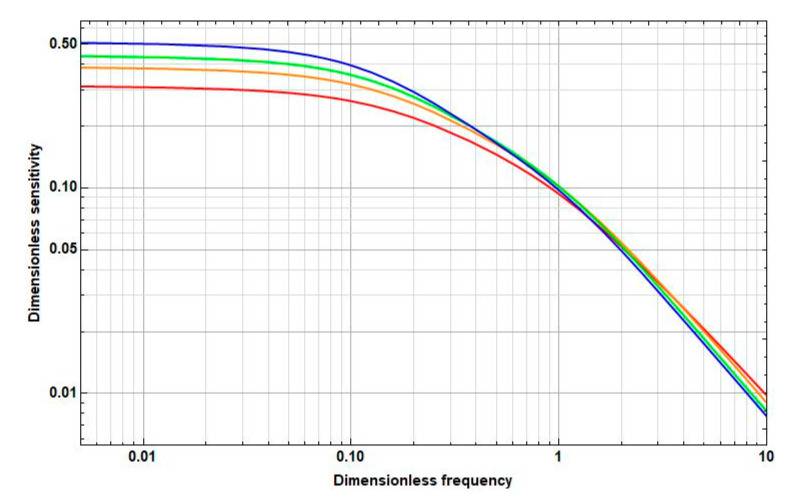
Frequency dependence of the sensitivity at different distances between the cathodes (red line, $2{\tilde{l}}_{c}=0$; orange line, $2{\tilde{l}}_{c}=0.5$; green line, $2{\tilde{l}}_{c}=1$; blue line, $2{\tilde{l}}_{c}=2$).

The graphs of the frequency dependence of the sensitivity at $2{\tilde{l}}_{c}=0$, $2{\tilde{l}}_{c}=0.5$, $2{\tilde{l}}_{c}=1$, and $2{\tilde{l}}_{c}=2$ are presented in Figure 9.

Figure 9 shows that the dependence of the sensitivity on the frequency becomes more uniform with a decrease in the intercathode distance. As a result, the upper cutoff frequency of the electrochemical cell increases with decreasing intercathode distance and becomes maximum at zero distance between the cathodes.

#### 3.3.3. The Experimental Validation

**Figure 10:**
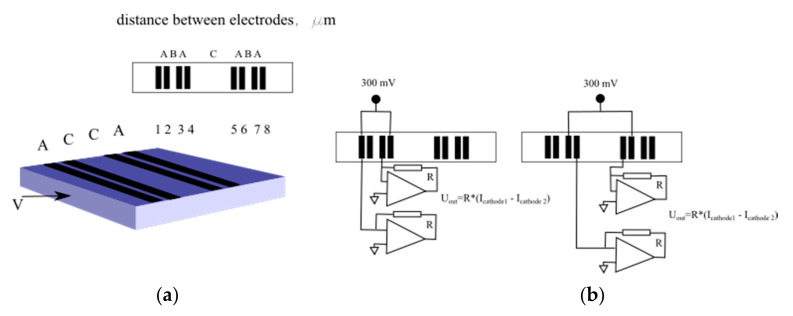
The experimental sample design (**a**) and two methods for powering and signal readout (**b**).

The experimental sample of the sensor with planar sensitive element described in [22] was used for the experimental validation of the model. The microstructure of the sensitive element was manufactured based on the silicon technology. The cell has eight electrodes 20 µm in width, combined in two groups deposited on a silicon plate. Two samples with different electrode distance were produced. The interelectrode distances for the said stricture are presented in Figure 10 (left) and in Table 1. The plate with the electrodes was glued on one of two non-conductive plates with the plates placed parallel to each other with 100 µm gap between them. In the experimental samples, the housings were produced so that the said gap between the plates worked as a channel for liquid motion under the influence of external acceleration. Rubber membranes were fixed on the ends of the channel, and a magnet was glued to one of the membranes and placed inside a coil, this building a calibration mechanism. Other details of the design as well as the description of the measurements using the excitation coil can be found in [22]. It should be noted that the channel sizes used in this design are not optimal for a practical converting structure, since a significant part of the flow is far from the electrodes and does not participate in the signal conversion. Meanwhile, the results obtained with its help are quite suitable for experimental verification of the developed model.

The electrodes of the experimental sample were connected to the power source and the signal conditioning electronics. The output signal $I_{out}$ was the difference between the currents passing through two cathodes. Generally, for this setup, the $I_{out}\;$ could be presented as the following:

(32) $$I_{out}/I_{coil}=W_{mech}W_{el-chem}K$$

Here, $I_{coil\;}$ is the excitation current passing in the coil; K  is the coil force constant; $\;W_{mech}$ is the transfer function for the mechanical subsystem, which characterizes the conversion of the force produced by the excitation coil into the pressure difference across the ends of the channel; and $W_{el-chem}\;$ is the transfer function of electrochemical cell.

Two methods of the electrode’s connection are shown in Figure 10 (right). Different connection methods can be seen to correspond to the electrochemical conversion cells with different distances between cathodes. They are B for the configuration marked as “Configuration 1” and C for the “Configuration 2” shown in Figure 10 (right). Two samples were produced with different geometrical parameters, as shown in Table 1.

The relative electrochemical cell transfer function, which characterizes the influence of the intercathode distance at different frequencies, was found from the experimental data according to the following formula:

(33) $$W_{el-chem,\;relative}\equiv \frac{W_{el-chem,\;1}}{W_{el-chem,2}}=\frac{{\left(I_{out}/I_{coil}\right)}_{1}}{{\left(I_{out}/I_{coil}\right)}_{2}}$$

Here, Indices 1 and 2 correspond to Configurations 1 and 2 shown in Figure 10.

**Figure 11:**
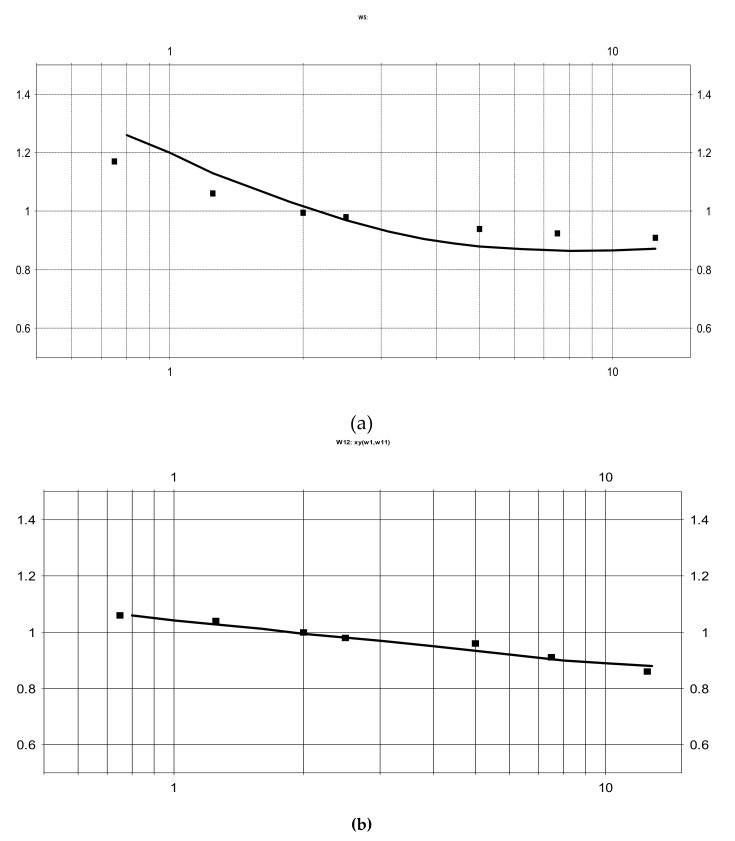
Relative frequency response for the cells with different distance between cathodes vs. dimensionless frequency. (**a**)—Sample 1; (**b**)—Sample 2.

The resulting experimental points are presented as squares in Figure 11. The modeling results for the same parameters are shown by a solid line. The upper curve corresponds to Sample 1 and the lower curve to Sample 2.

At low frequencies, the sensitivity for the configuration with a larger intercathode distance is noticeably higher, while at high frequencies above 1 Hz the situation is the opposite. This result agrees with the theoretical model presented in the current study and qualitatively validates the model presented in the paper.

For Sample 1 the experimentally measured relative transfer function changes 1.3 times in the given frequency range while the calculated value is 1.4. This accuracy in correspondence between the experimental and the theoretical data could be considered as a quite satisfactory taking into account the production tolerances in the experimental setup. Most important is the difference in the distance between the cathodes and adjusted anodes for Configurations 1 and 2 shown in Figure 10. The importance of this type of tolerances is a result of two factors: In Equation (1), there is strong dependence of the amplitude vs. frequency response on the distance between the cathode and the anode as noticed in previous publications [29,30], and, in Equation (2), the distance between the cathode and the anode is the smallest geometrical parameter for the system under consideration, thus it is the most sensitive to manufacturing tolerance.

For Sample 2, the experimental and theoretical data agree with each other with better accuracy, since the distance between cathodes and adjusted anodes is four times larger, which decreases the effect of production tolerances.

## 4. Discussion

This paper is the first one to study the effect of the space between the cathodes in a molecular-electron cell on the magnitude and distribution of the stationary background current and signal current. Therewith, the nature of that effect on stationary and signal (linear in speed) currents will be different.

In particular, an increase in the intercathode distance leads to an increase in the stationary current for all the studied parameters of the converting cell, as shown above.

**Figure 12:**
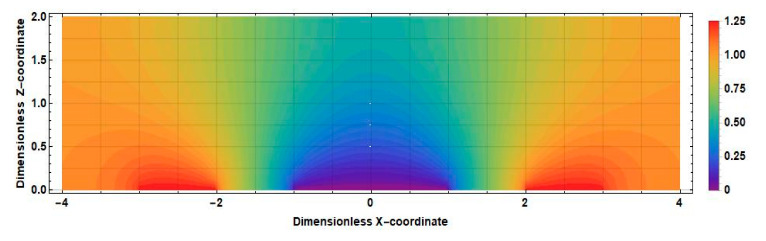
The distribution of the stationary concentration of the active component of an electrolyte solution in an electrochemical cell at $2{\tilde{l}}_{c}=0$.

**Figure 13:**
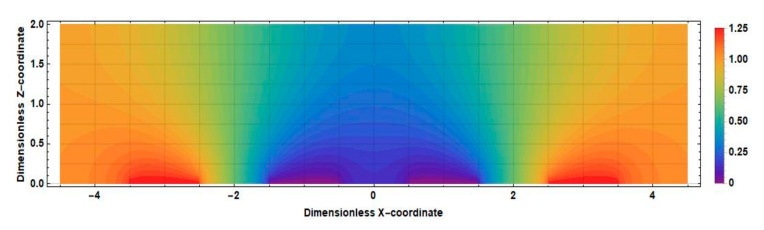
The distribution of the stationary concentration of the active component of an electrolyte solution in an electrochemical cell at $2{\tilde{l}}_{c}=1$.

The obtained result can be explained by comparing the distributions of the stationary concentration of the active component of the electrolyte solution at zero and nonzero distances between the cathodes shown in Figure 12 and Figure 13.

The main difference between the distributions shown in Figure 10 and Figure 11 is observed in the intercathode region. The figures show that the intercathode region is an additional source of the active component. The ions diffusion of ions of the active component of the electrolyte solution from this region leads to an increase in the current density in the near-cathode parts.

As for the signal current linear in speed, according to the calculations, the influence of the intercathode region on its value has different nature at low and high frequencies. At low frequencies, the conversion coefficient is higher for a cell with a large intercathode spacing, and vice versa at high frequencies.

The nature of such a nontrivial behavior of the signal current can be explained by referring to Equation (6). In its form, Equation (6) is a diffusion equation, where, on the right-hand side, the term -${\overset{\text{˜}}{v}}_{\overset{\text{˜}}{x}}\partial c_{0}\left(\tilde{x},\tilde{z}\right)/\partial \tilde{x}$, depending on its sign, represents either sources or drains for the active ions of an electrochemical cell. At the same time, at low frequencies, the value of the output current is affected by sources and drains located in a significant part of the cell volume. At high frequencies, diffusion to the electrodes is only possible from the volume of the cell directly adjacent to the cathodes and only ion sources located near the electrodes affect the output current.

**Figure 14:**
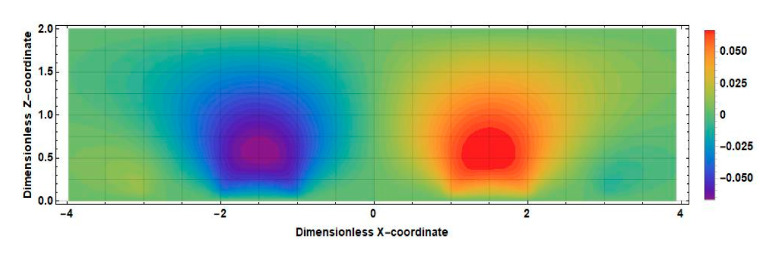
Distribution ${\overset{\text{˜}}{v}}_{\overset{\text{˜}}{x}}\frac{\partial c_{0}\left(\tilde{x},\tilde{z}\right)}{\partial \tilde{x}}$ in an electrochemical cell at $2{\tilde{l}}_{c}=0$.

**Figure 15:**
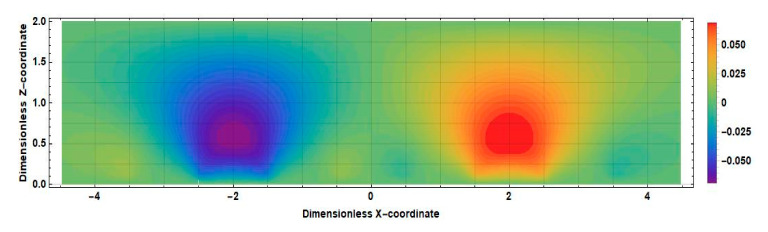
Distribution ${\overset{\text{˜}}{v}}_{\overset{\text{˜}}{x}}\frac{\partial c_{0}\left(\tilde{x},\tilde{z}\right)}{\partial \tilde{x}}$ in an electrochemical cell at $2{\tilde{l}}_{c}=1$.

The distribution ${\overset{\text{˜}}{v}}_{\overset{\text{˜}}{x}}\partial c_{0}\left(\tilde{x},\tilde{z}\right)/\partial \tilde{x}$ for the positive direction of speed is shown in Figure 14 ($2{\tilde{l}}_{c}=0$) and Figure 15 $\left(2{\tilde{l}}_{c}=1\right).$

Consider, for definiteness, the left half of the cell. Active ions, introduced into this region by a liquid flow, diffuse to the left anode and cathode. Accordingly, the sources in this area increase the current of the left cathode in absolute value, while the drains decrease it. In Figure 12, which corresponds to the case $2{\tilde{l}}_{c}=0$, in the whole half of the studied cell, it can be seen that ${\overset{\text{˜}}{v}}_{\overset{\text{˜}}{x}}\partial c_{0}\left(\tilde{x},\tilde{z}\right)/\partial \tilde{x}<0.$ The geometry with a nonzero intercathode distance (Figure 13) is distinguished by the presence and influence of the intercathode region corresponding to $-0.5<\tilde{x}<0.5$. Figure 13 shows that, for the left half of the cell, ${\overset{\text{˜}}{v}}_{\overset{\text{˜}}{x}}\partial c_{0}\left(\tilde{x},\tilde{z}\right)/\partial \tilde{x}>0\;$ at $\tilde{z}\leq 0.5$ and ${\overset{\text{˜}}{v}}_{\overset{\text{˜}}{x}}\partial c_{0}\left(\tilde{x},\tilde{z}\right)/\partial \tilde{x}<0$ at $\tilde{z}\geq 0.5.$ At the lowest frequencies, the entire cathode region contributes to the output cathode current, in most of which the sign of the quantity ${\overset{\text{˜}}{v}}_{\overset{\text{˜}}{x}}\partial c_{0}\left(\tilde{x},\tilde{z}\right)/\partial \tilde{x}\;$ is the same as in the rest of the studied half of the cell. Thus, for low frequencies, the influence of the intercathode region leads to an increase in the cathode current, while the cell with a nonzero intercathode distance has a higher conversion coefficient compared to the case of $2{\tilde{l}}_{c}=0$. For high frequencies, the situation is different: diffusion to the cathode is only possible from the region immediately adjacent to it, where for the left half of the cell ${\overset{\text{˜}}{v}}_{\overset{\text{˜}}{x}}\partial c_{0}\left(\tilde{x},\tilde{z}\right)/\partial \tilde{x\;}$ is negative. Accordingly, in this case, the influence of the intercathode space on the signal current is opposite to the influence of the rest of the cell, and the absolute value of the signal current decreases.

For the right half of the cell, the situation is similar with the difference: all ion sources are replaced by drains and vice versa, and the cathode current has the opposite sign at the same absolute value.

## 5. Conclusions

Summarizing the results obtained, we can conclude that the influence of the intercathode region size on the stationary and signal currents of the converting electrochemical cell is different. The stationary current decreases monotonically with a decrease in the intercathode distance with the constancy of other system parameters.

At the same time, the signal current decreases in the low-frequency region and grows in the high-frequency range.

A simple experimental setup proved the model.

The current dependences on the intercathode distance, which are found in this paper, have clear physical explanation. The explanation is based on inhomogeneity of a concentration gradient in the intercathode region. It leads to significant dependence of the signal current on the cathode edge close to the other cathode that depends on the external signal frequency.

Given the results obtained, practically speaking, it is advisable to reduce the intercathode distance. The fact is that, as a rule, at low frequencies, the conversion coefficient of the electrochemical cell is already quite high, and its further increase does not make any practical sense. On the other hand, an increase in sensitivity at high frequencies broadens the operating frequency range, and a decrease in stationary current reduces energy consumption.

Moreover, the results of the performed analysis and their physical interpretation can have a significant effect on the search for new engineering solutions that make it possible to increase the sensitivity of the conversion cell. For example, the data presented in Figure 3, Figure 4 and Figure 5 show that the signal currents in different parts of the electrodes behave differently, depending on the frequency of the external stimulus: they are added at low frequencies and subtracted at high frequencies, thus reducing the sensitivity of the converting element. In fact, this means that it is expedient to split the electrode into two or more parts, the currents from which must be summed up with frequency-dependent complex coefficients. Necessary specific technological solutions for the practical implementation of such ideas still need to be created.

## Figures and Tables

**Table 1:** Electrode cell dimensions.

	A, µm	B, µm	C, µm
Sample 1	5	20	100
Sample 2	20	20	80

## References

[1] Larkam C.W. (1965). Theoretical Analysis of the Solion Polarized Cathode Acoustic Linear, Transducer. J. Acoust. Soc. Am..

[2] Huang H., Agafonov V., Yu H. (2013). Molecular electric transducers as motion sensors: A review. Sensors.

[3] Agafonov V., Neeshpapa A., Shabalina A., Beer M., Kougioumtzoglou I.A., Patelli E., Au I.S.-K. (2015). Electrochemical Seismometers of Linear and Angular Motion. Encyclopedia of Earthquake Engineering SE–403–1.

[4] Koulakov I., Jaxybulatov K., Shapiro N.M., Abkadyrov I., Deev E., Jakovlev A., Kuznetsov P., Gordeev E., Chebrov V. (2014). Symmetric caldera-related structures in the area of the Avacha group of volcanoes in Kamchatka as revealed by ambient noise tomography and deep seismic sounding. J. Volcanol. Geotherm. Res..

[5] Liu C., Hua Q., Pei Y., Yang T., Xia S., Xue M., Le B.M., Huo D., Liu F., Huang H. (2014). Passive-source ocean bottom seismograph (OBS) array experiment in South China Sea and data quality analyses. Chin. Sci. Bull..

[6] Gorbenko V.I., Zhostkov R.A., Likhodeev D.V., Presnov D.A., Sobisevich A.L. (2017). Feasibility of using molecular-electronic seismometers in passive seismic prospecting: Deep structure of the Kaluga ring structure from microseismic sounding. Seism. Instrum..

[7] Agafonov V.M., Egorov E.I., Rice C.E. Closed-loop frequency MET geophone-operating principles and parameters. Proceedings of the 72nd European Association of Geoscientists and Engineers Conference and Exhibition 2010: A New Spring for Geoscience. Incorporating SPE EUROPEC 2010.

[8] Antonov A., Shabalina A., Razin A., Avdyukhina S., Egorov I., Agafonov V. (2017). Low-frequency seismic node based on molecular-electronic transfer sensors for marine and transition zone exploration. J. Atmos. Ocean. Technol..

[9] Zaitsev D., Agafonov V., Egorov E., Avdyukhina S. Broadband MET Hydrophone. Proceedings of the EAGE Conference & Exhibition.

[10] Egorov E.V., Shabalina A.S., Zaytsev D.L., Velichko G. Low Frequency Hydrophone for Marine Seismic Exploration Systems. Proceedings of the 5th International Conference on Sensors Engineering and Electronics Instrumentation Advances (SEIA’2019).

[11] Kostylev D.V., Bogomolov L.M., Boginskaya N.V. (2019). About seismic observations on Sakhalin with the use of molecular- electronic seismic sensors of new type. IOP Conf. Ser. Earth Environ. Sci..

[12] Kapustian N., Antonovskaya G., Agafonov V., Neumoin K., Safonov M. (2013). Seismic monitoring of linear and rotational oscillations of the multistory buildings in Moscow.

[13] Yudahin F.G., Kapustian N., Egorov E., Klimov A. (2012). An investigation of an external impact conversion into the strained rotation inside ancient boulder structures. Seismic Behaviour and Design of Irregular and Complex Civil Structures.

[14] Zaitsev D., Egor E., Shabalina A. (2019). High resolution miniature MET sensors for healthcare and sport applications. Proc. Int. Conf. Sens. Technol. ICST.

[15] Liang M., Huang H., Agafonov V., Tang R., Han R., Yu H. (2020). Molecular Electronic Transducer Based Tilting Sensors. Int. Conf. Micro Electro Mech. Syst..

[16] Agafonov V.M., Krishtop V.G. (2004). Diffusion Sensor of Mechanical Signals: Frequency Response at High Frequencies. Russ. J. Electrochem..

[17] Li G., Sun Z., Wang J., Chen D., Chen J., Chen L., Xu C., Qi W., Zheng Y. (2018). A Flexible Sensing Unit Manufacturing Method of Electrochemical Seismic Sensor. Sensors.

[18] Deng T., Chen D., Wang J., Chen J., He W. (2014). A MEMS Based Electrochemical Vibration Sensor for Seismic Motion Monitoring. Microelectromech. Syst. J..

[19] Yang D., Pan L., Mu T., Zhou X., Zheng F. (2017). The fabrication of electrochemical geophone based on FPCB process technology. J. Meas. Eng..

[20] Agafonov V., Egorov E. (2016). Influence of the electrical field on the vibrating signal conversion in electrochemical (MET) motion sensor. Int. J. Electrochem. Sci..

[21] Agafonov V., Egorov E. (2016). Electrochemical accelerometer with DC response, experimental and theoretical study. J. Electroanal. Chem..

[22] Agafonov A., Shabalina Ma D., Krishtop V. (2019). Modeling and experimental study of convective noise in electrochemical planar sensitive element of MET motion sensor. Sensors Actuators A Phys..

[23] Liang M., Huang H., Agafonov V., Tang R., Han R., Yu H. Molecular electronic transducer based planetary seismometer with new fabrication process. Proceedings of the IEEE International Conference on Micro Electro Mechanical Systems (MEMS).

[24] Krishtop V.G., Agafonov V.M., Bugaev A.S. (2012). Technological principles of motion parameter transducers based on mass and charge transport in electrochemical microsystems. Russ. J. Electrochem..

[25] Krishtop V.G. (2019). Technology and application o f electrochemical motion sensors. Adv. Mater. Proc..

[26] Thomas-Alyea J., Newman K.E. (2012). Electrochemical Systems.

[27] Xu Y., Lin W.J., Gliege M., Gunckel R., Zhao Z., Yu H., Dai L.L. (2018). A Dual Ionic Liquid-Based Low-Temperature Electrolyte System. J. Phys. Chem. B.

[28] Agafonov V. (2018). Modeling the Convective Noise in an Electrochemical Motion Transducer. Int. J. Electrochem. Sci..

[29] Zhevnenko D.A., Vergeles S.S., Krishtop T.V., Tereshonok D.V., Gornev E.S., Krishtop V.G. The simulation model of planar electrochemical transducer. Proceedings of International Conference on Micro- and Nano-Electronics 2016.

[30] Sun Z., Li G., Chen L., Wang J., Chen D., Chen J. (2017). High-sensitivity electrochemical seismometers relying on parylene-based microelectrodes. Eng. Mol. Syst..

